# Etiology and pharmacological treatment of delirious syndrome

**DOI:** 10.1192/j.eurpsy.2023.822

**Published:** 2023-07-19

**Authors:** J. Hamidović, L. Dostović Hamidović, S. Haskic, E. Prljača, A. Brigić, M. Mešanović

**Affiliations:** 1 Department od Psychiatry, University Clinical Center Tuzla, Tuzla; 2 Department od Pediatrics, University Clinical Center Tuzla, Tuzla; 3 Public Health Center, Čelić; 4 University Clinical Center Tuzla, Tuzla

## Abstract

**Introduction:**

Patients in psychiatric department, especially in the intensive care unit, often develop delirium syndrome, which leads to a high risk of morbidity and mortality. The etiology is multifactorial. The most common causes are alcoholism and dementia. Pharmacological treatment of delirious syndrome is the most important part of the treatment, which includes various psychopharmaceuticals that are effective both in the treatment of delirium and in improving cognitive functions. Haloperidol is the drug of first choice and from atypical antipsychotics, the most commonly used are risperidone and olanzapine. Benzodiazepines are used in the treatment of delirium tremens.

**Objectives:**

The objective of the work is to determine the most common cause of delirious syndrome and the treatment of those patients.

**Methods:**

We analyzed 52 patients who were treated for delirious syndrome at the Department of Psychiatry , University Clinical Center Tuzla, Bosnia and Herzegovina in the period from January 1, 2019. until June 1, 2022. Data were taken from medical records and the hospital information system.

**Results:**

The total number of patients was 52 and 23 (44.23%) were treated for delirium tremens, and the rest were treated for delirium syndrome of another cause. The most common other causes were dementia in 21 (40.38%) patients, followed by sepsis, infectious syndrome and tumors in 6 (11.53%) patients, and cerebrovascular cause in 2 (3,84%) patient. In a therapeutic approach of delirious syndrome, all patients with delirium tremens were treated with benzodiazepines: 11 (47.82%) patients with diazepam monotherapy, then diazepam and promazine 7 (30.43%) patients, diazepam and haloperidol 3 (13,04%) patients, and diazepam, olanzapine and haloperidol 2 (8.69%). In the therapy of other delirious syndromes, 11 (37.93%) patients were treated with risperidone, haloperidol 8 (27.58%), promazine 3 (10.34%), quetiapine 4 (13.79%), and olanzapine, clozapine and aripiprazole 1 patient each (3.44%). It is important to point out that there was no fatal outcome in the processed sample of patients.

**Conclusions:**

The most common etiological cause of delirious syndrome is the consequence of alcohol withdrawal. Delirium superimposed on dementia is the second most common. The priority of treatment is focused on pharmacological treatment. Atypical antipsychotics (risperidone) are most often used. Haloperidol is the second most common. Benzodiazepine (diazepam) was most often used in the treatment of delirium tremens.

**Disclosure of Interest:**

None Declared

